# TBL1XR1-RARB融合基因阳性儿童急性早幼粒细胞白血病1例

**DOI:** 10.3760/cma.j.issn.0253-2727.2022.08.018

**Published:** 2022-08

**Authors:** 亚芥 张, 巧茹 黎, 丹娜 林, 莉 吴, 明 程, 露露 黄, 肖蓉 赖, 娟 资, 旭 廖, 宇婷 袁, 文鑫 张, 瑞清 王, 丽华 杨

**Affiliations:** 1 南方医科大学珠江医院小儿血液科，广州 510280 Department of Pediatrics, Zhujiang Hospital of Southen Medical University, Guangzhou 510280, China; 2 中山市人民医院普通儿科，佛山 528000 Department of Pediatrics, Zhongshan People's Hospital, Foshan 528000, China

患儿，男，2岁10个月，因“发热3 d，发现血常规异常2 d”于2021年3月14日就诊于南方医科大学珠江医院小儿血液科。入院时血常规：WBC 24.82×10^9^/L，中性粒细胞占85.5％，HGB 72 g/L，PLT 105×10^9^/L。凝血功能：凝血酶原（PT）14.9 s，部分凝血活酶时间（APTT）29.6 s，纤维蛋白原（Fg）1.22 g/L，D-二聚体3.07 mg/L。血生化检查：乳酸脱氢酶（LDH）1718.3 IU/L。骨髓细胞形态学：考虑急性早幼粒细胞白血病（APL）。染色体核型正常。流式细胞术检测免疫表型：异常细胞占有核细胞总数约84.6％，符合急性髓系白血病（AML）免疫表型。头颅磁共振（MRI）增强扫描提示：双侧侧脑室后角及枕角旁小片状异常信号，脑室系统稍扩大；双侧额裂骨、颅底骨质及下颌骨支不均匀增厚伴明显强化，不排除白血病浸润。胸腹部CT：拟右肺间质性炎症及少许炎症灶；左侧第7后肋骨质破坏并软组织肿块形成，考虑白血病浸润可能。2021年3月16日，根据骨髓细胞形态学初步诊断为APL，按华南儿童癌症协作组（SCCCG）APL2020高危方案，开始口服全反式维甲酸（ATRA，20 mg·m^−2^·d^−1^）和复方黄黛片（RIF，135 mg·kg^−1^·d^−1^）治疗，2021年3月20日，骨髓标本PML-RARA融合基因PCR定量检测为阴性。骨髓标本PML-RARA融合基因荧光原位杂交（FISH）检测为阴性。骨髓标本AML相关基因突变检测：KARS p.Q61H变异（变异频率12.15％）、NARS p.G13V变异（变异频率0.9％）、TBL1XR1 c.427+1G>A变异（变异频率2.12％）。修正诊断为PML-RARA融合基因阴性的APL，遂送检初始骨髓标本，加做白血病相关基因转录组测序（RNA-seq）。2021年4月2日，评估APL方案的疗效，外周血涂片未见幼稚细胞。骨髓细胞形态学提示原始早幼粒细胞占4％，骨髓多参数流式细胞术（MFC）检测微小残留病（MRD）为1.9％，骨髓细胞形态学示完全缓解。复查头颅MRI增强扫描及肺部CT扫描，以上髓外浸润病灶消失。2021年4月7日骨髓白血病相关基因RNA-seq定量检测结果报告：TBL1XR1-RARB融合基因36.54％（目的基因/内参基因36.54％）。2021年4月13日（化疗第27天），修正诊断为TBL1XR1-RARB融合基因阳性APL，按照SCCCG-APL2020方案的标准，非RARA基因重排的APL应按照非APL的AML方案化疗，改按华南儿童AML（华南AML-15）诊疗方案化疗，FLAG（氟达拉滨、阿糖胞苷、G-CSF）+去甲氧柔红霉素（IDA）方案诱导治疗2个疗程，HA（高三尖杉酯碱+阿糖胞苷）、EA（依托泊苷+阿糖胞苷）、DA（柔红霉素+阿糖胞苷）方案各巩固治疗1个疗程，第1个疗程诱导治疗后骨髓MRD（MFC）1.8％，融合基因定量阴性，之后每个疗程后检测骨髓MRD（MFC）和融合基因均为阴性。诱导化疗第1疗程结束后合并粒细胞缺乏伴感染，经抗感染治疗控制；诱导化疗第2疗程结束后合并粒细胞缺乏伴铜绿假单胞菌败血症和侵袭性曲霉菌肺炎，经抗感染及对症治疗后治愈；巩固化疗1个疗程结束后合并肺炎克雷伯菌败血症，经抗感染及对症治疗后治愈；巩固治疗第2、3疗程结束后均合并粒细胞缺乏，但无感染发生。

对于该类型的APL，目前尚无统一的治疗方案，且考虑到该例患儿在按APL方案化疗27 d后（改为华南AML-15方案治疗前），骨髓TBL1XR1-RARB融合基因水平由36.54％降至2.38％。因此，于2021年9月29日结束化疗后，给予为期半年的ATRA联合RIF维持化疗：ATRA 15 mg·m^−2^·d^−1^，用1周停1周；RIF 50 mg·kg^−1^·d^−1^，用2周停2周。2022年4月1日结束维持治疗，维持治疗期间每3个月复查骨髓MRD（MFC）和TBL1XR1-RARB融合基因水平，均为阴性。目前患儿生长发育良好。

